# Pushing the frontiers in the fight against antimicrobial resistance: the potential of fecal and maggot therapies

**DOI:** 10.2144/fsoa-2023-0089

**Published:** 2023-08-29

**Authors:** Bashar Haruna Gulumbe, Abdulrakib Abdulrahim

**Affiliations:** 1 Department of Microbiology, Faculty of Science, Federal University, Kalgo, Birnin Kebbi, PMB, 1157, Nigeria

**Keywords:** antimicrobial resistance, biotherapeutics, fecal microbiota transplantation, gut microbiome, maggot debridement therapy

## Abstract

The escalating crisis of antimicrobial resistance (AMR) warrants innovative therapeutic strategies. Fecal microbiota transplantation (FMT) and maggot debridement therapy (MDT) represent paradigm-shifting approaches, leveraging biological systems to mitigate AMR. FMT restores a healthy gut microbiome, providing a biotherapeutic counter to pathogenic bacteria, thereby reducing reliance on traditional antibiotics. Conversely, MDT, a form of bio-debridement, utilizes the antimicrobial secretions of maggots to cleanse wounds and eliminate resistant bacteria. Despite the promise these therapies hold, their broader clinical adoption faces multifaceted challenges including the need for rigorous scientific substantiation, standardized protocols, deepened understanding of mechanisms of action, and surmounting regulatory and public acceptance barriers. However, their potential integration with precision medicine could revolutionize disease management, particularly with antibiotic-resistant infections.

## Plain language summary

The rising problem of drug-resistant infections calls for new treatment methods. Fecal microbiota transplantation (FMT) and maggot debridement therapy (MDT) offer innovative solutions. FMT uses healthy gut bacteria to fight harmful ones, while MDT employs maggots to clean wounds and kill resistant bacteria. Although promising, these methods face challenges like a lack of understanding, standardization and public acceptance. Their potential, however, could transform how we manage antibiotic-resistant diseases.

## Tweetable abstract

Fecal microbiota transplantation (FMT) and maggot debridement therapy (MDT) present paradigm-shifting strategies to tackle antimicrobial resistance (AMR). Offering biological counters to pathogenic bacteria & antibiotic reliance. Promise & challenges ahead. #AMR #FMT #MDT.

Antimicrobial resistance (AMR) is a formidable challenge that the global health community currently faces [1]. This escalating crisis threatens to destabilize the foundations of modern medicine [1]. Recognizing the severity of this issue, the WHO has classified AMR among the top ten global public health threats [2]. The persistent emergence of multi-drug-resistant microorganisms has led to a situation where many of our existing antibiotics are losing their effectiveness [1,3]. Consequently, drug-resistant infections are now once again a leading cause of death worldwide [1]. This problem is further exacerbated by the concerning dearth of new antimicrobial agents in the development pipeline [4]. This alarming scenario necessitates an immediate exploration of innovative and unconventional therapeutic strategies to counteract the impending crisis [4,5].

In response to this urgent need, this piece examines two unconventional yet potentially promising therapeutic approaches: fecal microbiota transplantation (FMT) and maggot therapy. FMT is a procedure which has been used since third-century China [6], involves the transfer of gut microbiota from a healthy individual to a patient [7]. The approach has demonstrated potential in restoring gut microbial balance and combating certain types of antibiotic-resistant infections [8]. Conversely, maggot therapy, a practice with historical roots and recent resurgence, capitalizes on the unique abilities of fly larvae to clean wounds, remove necrotic tissue and secrete antimicrobial substances [9,10]. This process aids in wound healing and could potentially help combat resistant pathogens [11].

This paper aims to provide a comprehensive overview of these unconventional therapies, examining their historical context, the mechanisms underlying their action, their potential clinical applications and the challenges that need to be addressed to facilitate the transition of these therapies from the laboratory to the clinic. As we strive to push the boundaries in our battle against AMR, we must not hesitate to explore unconventional avenues. However, the journey from bench to bedside is fraught with challenges. The safety and efficacy of these therapies need to be thoroughly evaluated through rigorous scientific research [7,11]. Regulatory frameworks need to be established to ensure that these therapies are administered safely and ethically. And perhaps most importantly, the stigma associated with these unconventional therapies needs to be addressed. Education and awareness are key to overcoming these barriers and facilitating the acceptance of these therapies.

As we stand on the precipice of a post-antibiotic era, it is imperative that we explore all possible avenues in our fight against AMR. The potential of FMT and maggot therapy represents a beacon of hope in this daunting scenario. Through rigorous research and open-minded exploration, we may yet turn the tide in our battle against antimicrobial resistance. The understanding and application of these therapies could open up new avenues in the fight against AMR, providing us with additional tools in our therapeutic arsenal.

## The microbiome & antimicrobial resistance

The vast and intricate assemblage of microorganisms that reside within the human body, known as the human microbiome, has become an increasingly significant area of focus in medical research [12,13]. This microbial community, predominating in the gut but also populating other regions of the body, has been found to play a central role in a multitude of health outcomes [12,13]. In particular, the concept of dysbiosis, an imbalance or disruption in the composition of the microbiome has been implicated in a spectrum of health issues [14]. These range from metabolic to immune-related disorders [15], and more recently, to the burgeoning concern of AMR [16].

Understanding the relationship between the microbiome and AMR involves considering two interconnected facets. The first perspective regards the gut microbiota as a potential reservoir of antimicrobial resistance genes, known collectively as the resistome [17]. This vast genetic resource can be disseminated among bacteria through the process of horizontal gene transfer [17]. This mechanism allows for the sharing of genetic material between different bacterial species, including commensal and pathogens, thus facilitating the proliferation of resistance genes throughout the microbial community [17]. The second perspective focuses on the susceptibility of an individual to infections by antibiotic-resistant pathogens in the context of dysbiosis. A balanced and diverse microbiome plays an instrumental role in maintaining health by outcompeting potential pathogens for resources and colonizing space, producing antimicrobial substances, and promoting a robust immune response [16,18]. Any disruption in this balance (dysbiosis) compromises these protective mechanisms, creating a conducive environment for antibiotic-resistant organisms to establish and flourish [16].

Given these dynamics, the use of FMT has been proposed as a potentially effective intervention for restoring a balanced microbiome and thereby helping to curb AMR [16,19]. The premise of FMT involves the transfer of fecal matter which is rich in commensal bacteria from a healthy individual to a patient [19]. The objective is to re-establish a diverse community of beneficial bacteria in the patient's gut, thereby restoring the protective functions of a balanced microbiome and enhancing resilience against antibiotic-resistant pathogens [16,19].

The potential for FMT as a tool in the fight against AMR is a relatively new area of research that is beginning to gain recognition. FMT has been conventionally used for the treatment of recurrent *Clostridioides difficile* infection, a condition known to be linked to dysbiosis [8]. However, the potential therapeutic applications of FMT may extend beyond this particular infection. The gut microbiome's role in health extends far beyond its local environment, influencing distant organs and systems through various mechanisms, including immune modulation, metabolic regulation and production of bioactive compounds [12,15,18]. Therefore, the impact of dysbiosis and by extension, the potential benefits of microbiome restoration through FMT may be far-reaching. This potential is beginning to be explored in various areas of medicine, from metabolic to neurological disorders [12], and notably in the context of AMR [16,20].

While the precise mechanisms by which FMT could help combat AMR are not fully understood, preliminary research indicates several possible avenues. For instance, the reintroduction of a diverse microbiota through FMT could outcompete resistant pathogens for resources and space, limit their proliferation, and reduce the likelihood of resistant infections [16,20]. Furthermore, certain members of a healthy microbiota could potentially degrade antibiotics or sequester them [21,22], reducing the selective pressure that drives the evolution of resistance. Therefore, harnessing the microbiome's protective power through FMT may represent promising strategy in the global fight against AMR.

## Fecal microbiota transplants: a frontier in microbiome therapy

In the realm of combating AMR, FMT stands as a promising frontier in microbiome therapy. Its proven efficacy in treating Recurrent *Clostridium difficile* Infection (rCDI), a condition intrinsically tied to antibiotic usage and resistance [23,24], underscores its potential in this arena. The role of the gut microbiota as both a potential reservoir for resistance genes and a critical line of defence against resistant pathogens highlights the potential of FMT as a two-pronged approach: reducing the spread of resistance by diluting the resistome and enhancing resistance against colonization by resistant pathogens [16,20]. Since the first randomized clinical trial in 2013 showed that FMT outperformed antibiotics in treating rCDI [25]. FMT's potential is extending into other conditions associated with dysbiosis, including inflammatory bowel disease [26], irritable bowel syndrome [27], and metabolic disorders [28]. The hypothesis that a healthy, balanced microbiome can alleviate these conditions provides a robust foundation for further research into FMT's potential applications.

In a recent multicenter phase I trial, the potential of FMT was explored in combination with PD-1 inhibitors nivolumab or pembrolizumab in previously untreated patients with advanced melanoma [23]. The study found FMT from healthy donors to be safe in the first-line setting, with no significant grade 3 adverse events from FMT alone [23]. More interestingly, a separate study has demonstrated FMT's potential in reducing the abundance of antibiotic-resistant genes in patients with dysbiosis and successfully decolonizing multidrug-resistant organisms [16,20,29]. However, these studies represent only the initial stages of exploration. Thus, rigorous long-term studies, and comprehensive analyses are necessary to elucidate the mechanisms underlying FMT's effects and to assess its long-term safety and efficacy. Similarly, despite its potential, the translation of FMT into clinical practice faces several challenges [7,25,30]. The development of standard protocols, quality control measures, and a better understanding of FMT's long-term impact are all essential to ensure patient safety and treatment efficacy [7]. A move toward ‘targeted’ FMT, involving defined mixtures of beneficial bacteria, could offer more precise, customizable treatment options and address some of the current challenges. In a recent publication, Ianiro and colleagues [31] were able to predict the donors with the highest potential to shape the recipient's microbial composition toward specific features such as increased species richness, a decreased proteobacterial richness or an increased cumulative abundance of bacteria associated with favorable cardiometabolic health for example. Together with a better identification of disease- and health-associated microbial features for each specific disease, this approach could lead to the development of therapeutic FMT strategies based on the selection of the recipient-specific optimal donor within a set of available donors, or the ad hoc assembly of strain consortia.

FMT is positioning itself as a novel strategy in the fight against AMR [16,20]. Its ability to restore a healthy microbiome and potentially reduce the spread of resistance presents a unique opportunity in this context. While the road to clinical application may be fraught with challenges, the potential rewards justify the continued exploration of FMT as a frontier in microbiome therapy.

## Maggot debridement therapy: An old approach with new potential

The concept of using maggots for therapeutic purposes, while repulsive to some, is not new. The healing properties of maggots have been acknowledged since antiquity, with their use in wound debridement dating back centuries [10,11]. Now, with the rising threat of AMR, MDT is being revisited as a potential adjunct or alternative to conventional antimicrobial therapies [32,33]. The modus operandi of MDT involves the application of sterile larvae of the green bottle fly, *Lucilia sericata*, to necrotic or infected wounds [11,32]. The larvae consume necrotic tissue, aiding in wound debridement, while secreting antimicrobial substances that can inhibit the growth of pathogenic bacteria, including resistant strains [33]. By selectively devouring necrotic tissue and sparing healthy tissue, maggots provide a form of biological debridement that can be particularly beneficial in chronic, non-healing wounds [32].

MDT's potential in the context of AMR lies in its dual action: physical wound debridement coupled with the secretion of antimicrobial substances. The enzymes secreted by the maggots can break down biofilms (complex bacterial communities) often found in chronic wounds that are notoriously resistant to antibiotics [33]. The antimicrobial compounds produced by maggots also display broad-spectrum activity [33,34], potentially providing a means of circumventing conventional antibiotic resistance.

The application of MDT has proven effective in managing chronic wounds such as diabetic foot ulcers [35], venous leg ulcers [36] and pressure sores [37] as well as other kinds of wounds where traditional therapies often fail [11]. Furthermore, some studies have shown that wounds treated with MDT have lower rates of bacterial colonization, including by antibiotic-resistant strains, supporting MDT's potential role in the fight against AMR. For example, in a 2021 study focused on the effect of bacterial colonization and MDT on wound healing in chronic venous leg ulcers, Sirekbasan *et al.* [38] found significant results. The microorganisms isolated from the wounds showed a notable decrease immediately following the initial MDT session.

Biofilms are complex assemblies of microbial communities shielded within a self-generated matrix, rendering them resilient to traditional antimicrobial interventions [39]. In addition to their inherent phenotypic recalcitrance to traditional antibiotics, biofilms provide environments that foster and fuel AMR [40]. Biofilms enable exposure to subinhibitory concentrations of antibiotics coupled with high cell densities, increased genetic competence and the accumulation of genetic elements or uptake of resistance genes. Horizontal transfer of resistance genes via conjugation is the sole mechanism identified for biofilm-mediated resistance-gene acquisition, with several studies showing it to be more efficient in bacterial biofilms as compared with planktonic cells [39]. MDT addresses this resistance through the active degradation of the biofilm structure, facilitated by enzymes such as proteases and lipases secreted by the larvae [40]. Coupled with the mechanical disruption caused by the larvae, the underlying bacteria are exposed and rendered more vulnerable to antimicrobial treatments [40]. This distinctive, dual-action approach enhances the efficacy of existing therapies and illuminates new pathways to subvert the intricate mechanisms of resistance, positioning MDT as a vital component in the multifaceted battle against AMR.

However, as with any therapy, MDT is not without its challenges. Patient acceptance of MDT can be a significant hurdle in its widespread adoption. The utilization of maggots in medical treatment often elicits a natural repulsion or discomfort among many individuals. This psychological barrier may stem from cultural perceptions, personal phobias, or general unease with the unconventional nature of the therapy [11,37]. The challenge, then, lies not just in demonstrating the efficacy and safety of MDT but also in educating patients and healthcare providers to overcome these instinctive reactions. This calls for a comprehensive approach, integrating scientific evidence with empathetic communication and potentially involving counseling or other support mechanisms to facilitate acceptance [11,37]. Additionally, standardized protocols and guidelines for MDT use are still in development, reflecting the therapy's relatively novel position in the medical landscape. The lack of universally accepted procedures can lead to inconsistencies in application and outcomes, creating challenges in both clinical practice and research [11]. Despite these challenges, the potential benefits of MDT in the era of rising AMR cannot be overlooked. As research advances, a better understanding of MDT's mechanisms of action and its potential to complement or replace conventional antimicrobial therapies could pave the way for its wider acceptance and use. As such, MDT represents another promising frontier in the ongoing battle against AMR.

## Converging paths: FMT & MDT in the fight against AMR

The unconventional yet promising modalities of FMT and MDT, despite being disparate in their origins and applications, intersect in their potential to alleviate the AMR crisis. Each harnesses biological mechanisms in their approach, either by reinstating a protective microbiome or employing a biological debridement technique with additional antimicrobial effects. This shared principle of utilising biological means to target pathogenic bacteria could potentially reduce the dependency on antibiotics, thereby mitigating the proliferation of resistance. These therapeutic strategies represent a paradigm shift in the management of microbial resistance, symbolising the potential of intricate biological systems to transform healthcare. Their broader implications suggest a profound impact on the overall approach to disease management and health promotion. As the medical field moves toward precision medicine, the potential to customise these therapies to individual patients' needs presents an exciting frontier. For instance, the prospect of targeted FMT, using defined combinations of beneficial bacteria, and the potential fine-tuning of MDT to cater to specific wound environments, signal a future where these therapies can be personalized.

In essence, FMT and MDT provide potential innovative pathways in the ongoing battle against AMR. As our comprehension of complex biological systems deepens, these therapies could pave the way for a more sustainable and efficient approach to managing AMR. While the path to realising their full potential will require dedicated scientific investigation, the initial promise warrants continued exploration and investment in these research areas.

## Pros, cons & future challenges of FMT & MDT in the fight against AMR

AMR continues to pose a formidable challenge to global health, necessitating innovative therapeutic strategies [41]. Two intriguing prospects in this battle are FMT MDT. This examination delineates the multifaceted advantages and disadvantages of both approaches, paving the way for more informed decisions in the quest to combat AMR. Table 1 offers an extensive overview of the primary benefits and drawbacks associated with FMT and MDT.

**Table 1.** An overview of the benefits and drawbacks associated with FMT and MDT.

Therapy	Pros	Cons
Fecal microbiota transplantation	1. Effectiveness in recurrent *C. difficile* Infections 2. Potential in other gut disorders 3. Personalized treatment options 4. Potential metabolic benefits 5. Potential in modulating immune responses 6. Lower antibiotic usage 7. enhancing gut flora diversity	1. Limited understanding of long-term effects 2. Potential transmission of undetected pathogens 3. Variability in donor stool quality 4. Lack of standardized protocols 5. Ethical considerations 6. Potential unwanted weight gain or metabolic changes 7. Risk of exacerbating existing conditions 8. Public perception and acceptance
Maggot Debridement Therapy	1. Proven efficacy in wound healing 2. Natural debridement mechanism 3. Anti-bacterial properties 4. Stimulation of wound healing factors 5. Reduced healing time 6. Limited side effects 7. Applicability to various wound types	1. Patient acceptance 2. Limited application 3. Potential allergic reactions 4. Aesthetic concerns 5. Requirement of specialized training 6. Inconsistent availability 7. Risk of secondary infection 8. Lack of standardized protocols

The advantages of FMT are anchored in its biological approach. Its effectiveness in recurrent *C. difficile* infections represents more than a treatment for a specific disease [6]; it demonstrates a broader potential to modulate the gut microbiome in ways that can benefit overall health. This could lead to applications in inflammatory bowel disease, obesity and even autoimmune diseases. The personalized nature of FMT allows for a tailored therapeutic strategy, catering to the unique microbial environment of individual patients [6]. However, the scientific understanding of FMT is still maturing. The long-term effects remain uncertain, and the potential for transmitting undetected pathogens is an ongoing concern. The lack of standardized protocols highlights the field's nascent stage, creating challenges in ensuring consistent treatment quality. Ethical considerations and public perceptions further complicate FMT's widespread adoption [6].

MDT's efficacy in wound healing is well-documented, offering a cost-effective and natural method of debridement. Its antibacterial properties provide a dual function, cleaning the wound while promoting healing [9,10]. This natural synergy aligns with a growing interest in more holistic, biologically-integrated medicine. Despite its therapeutic promise, MDT faces perceptual and practical barriers [35]. Acceptance by patients and healthcare providers may be challenging, given aesthetic concerns and the unconventional nature of the therapy. Specialized training requirements limit its accessibility, and inconsistent availability may hinder its routine use [11]. The lack of standardized protocols reveals an area in need of professional consensus to ensure safety and efficacy.

FMT and MDT in the context of AMR open promising avenues that are yet marked by a profound lack of comprehensive evidence. The advantages and limitations illuminated within this examination underscore a complex interplay between scientific potential and unresolved challenges. Our current understanding, although advancing, is nascent and overshadowed by the vastness of what remains uncharted. The field beckons for methodologically rigorous, systematic investigations that extend beyond anecdotal successes and delve into the core mechanisms, safety profiles and long-term outcomes. The guarded optimism surrounding FMT and MDT must be matched with a steadfast commitment to further research, embracing a multidisciplinary approach that fosters innovation, transparency, and a nuanced understanding of these novel interventions. This informed pursuit can turn experimental therapies into viable strategies in our fight against AMR, yet it demands we navigate with caution, guided by evidence and a recognition of the complexities involved.

## Regulatory challenges & considerations in advancing FMT & MDT

Emerging therapies such as FMT and MDT, promising as they are in combating AMR, bring with them a distinct set of regulatory challenges [34,42]. Their marked departure from traditional pharmaceuticals necessitates a careful reevaluation of existing regulatory frameworks to accommodate these novel interventions.

A prominent regulatory issue for FMT revolves around its classification. The designation of fecal material whether as a drug, a tissue, or a unique entity significantly influences the regulatory requirements it must meet [42,43]. This classification affects the nature of required clinical trials, applicable manufacturing standards, and permitted marketing and distribution strategies [42,43]. Ensuring the safety of FMT is another regulatory priority. While FMT has a proven safety record for rCDI treatment [30], its safety profile in other applications demands further exploration. Recognizing potential risks associated with disease transmission or other unforeseen adverse events is paramount [27]. Comprehensive donor screening and testing protocols, along with vigilant post-market surveillance systems, are integral to maintaining FMT safety [43].

MDT, though distinct, faces similar regulatory hurdles. As the therapy involves the introduction of living organisms (maggots) into open wounds, there are inherent safety and quality control concerns [9,11,35]. Regulatory considerations must address the production of sterile maggots, management of possible adverse reactions and standardization of treatment protocols [10,11].

Moreover, both FMT and MDT present challenges regarding the intersection of regulation and public perception is critical in determining patient acceptance of these therapies. Robust regulatory oversight that is transparent and clear can bolster public confidence in these treatments and increase their acceptance [44]. As these fields continue to evolve, ongoing dialogue among all stakeholders, scientists, clinicians, regulators, and patients will be vital to navigate these challenges and fully harness the potential of FMT and MDT in the fight against AMR.

## Conclusion

The escalating threat of AMR underscores the pressing need for innovative therapeutic strategies. The biological underpinnings of FMT and MDT offer novel approaches that may help circumvent the rise of resistance by reducing reliance on traditional antibiotics. For example, a recent study demonstrated FMT's potential as a first-line treatment in patients with advanced melanoma, supporting its broader applications beyond recurrent *C. difficile* infections. Conversely, the use of MDT has been shown to specifically target biofilms, which are key in AMR gene transmission. However, patient acceptance of MDT can be a significant hurdle, given the repulsion many feel toward maggots, and standardized protocols and guidelines for MDT use are still in development. Furthermore, the path to broader clinical adoption of these therapies is fraught with challenges, including a lack of deepened understanding of the precise mechanisms underlying their therapeutic effects, and the need for rigorous scientific substantiation, standardized procedural protocols, surmounting hurdles in regulatory landscapes and public acceptance. Successful advancement of these therapies will necessitate a coordinated multidisciplinary approach, involving the concerted efforts of clinicians, researchers, regulators and patients alike.

## Future perspective

Peering into the future, the intersection of FMT and MDT with precision medicine heralds intriguing prospects. The potential to customise these therapies to individual patients could precipitate a paradigm shift in the management of various health conditions, particularly those involving antibiotic-resistant infections. The introduction of a table delineating the multifaceted pros and cons of FMT and MDT provides a comprehensive overview to aid in future decisions. However, the transition from experimental therapies to routine clinical applications necessitates robust and multifaceted endeavors. A deeper understanding of the precise mechanisms underlying its therapeutic effects remains an area of ongoing exploration. While the benefits of MDT in wound healing and bacterial control are recognized, the specific biochemical and cellular processes that facilitate these outcomes are not fully elucidated. This gap in knowledge highlights the need for further scientific investigation to refine the methodology, enhance its efficacy and broaden its applicability in the fight against antimicrobial resistance. The formidable challenge posed by AMR is matched by the potential of innovative solutions to counter it, but there is still a pressing need for further research, especially considering the lack of substantial evidence supporting these therapies. As the field evolves, it is the hope that these therapies will offer fresh ammunition to curtail the burgeoning threat of AMR, contributing toward a more sustainable and effective strategy in managing infectious diseases. The potential benefits of FMT and MDT in the struggle against AMR warrant sustained investment in these research areas, as evidenced by the fact that drug-resistant infections are now once again a leading cause of death worldwide.

**Executive summary**

- Antimicrobial resistance (AMR) constitutes a global health emergency; fecal microbiota transplantation (FMT) and maggot debridement therapy (MDT) are pioneering therapeutic options to confront this mounting predicament.

**The microbiome & antimicrobial resistance**

- Delving into the biological foundations of FMT and MDT, these innovative methodologies may offer significant avenues to alleviate AMR, with implications for reducing reliance on traditional antibiotics.

**Fecal microbiota transplants: a frontier in microbiome therapy**

- FMT is emerging as a promising intervention against pathogenic bacteria; however, comprehensive scientific validation is vital for its wider clinical application.

**Maggot debridement therapy: an old approach with new potential**

- Through the utilization of maggots' antimicrobial secretions, MDT promises a unique wound treatment strategy; however, standardized application and public perception remain challenges.

**Converging paths: FMT & MDT in the fight against AMR**

- The potential synergy between these therapies could represent a paradigmatic transformation in the management of antibiotic-resistant infections.

**Pros, cons & future challenges of FMT & MDT in the fight against AMR**

- While heralding significant potential, these therapies must traverse a multifaceted landscape of challenges, encompassing scientific substantiation, procedural standardization, mechanistic comprehension and regulatory and public acceptance.
- The regulatory environment poses significant hurdles; successful implementation will require coordinated efforts across clinical, research, regulatory and patient communities.

**Future perspective**

- The integration of FMT and MDT with precision medicine foreshadows a radical shift in the management of infectious diseases, particularly those characterized by antibiotic resistance; yet, the transition from experimental to routine clinical applications will necessitate robust, multifaceted efforts.
